# Evolutionary Adaptation of the Thyroid Hormone Signaling Toolkit in Chordates

**DOI:** 10.3390/cells10123391

**Published:** 2021-12-02

**Authors:** Alfonso Esposito, Luca Ambrosino, Silvano Piazza, Salvatore D’Aniello, Maria Luisa Chiusano, Annamaria Locascio

**Affiliations:** 1 Computational Biology Unit, International Centre for Genetic Engineering and Biotechnology, ICGEB, 34149 Trieste, Italy; alfonso.esposito@icgeb.org (A.E.); silvano.piazza@icgeb.org (S.P.); 2 Department of Research Infrastructure for Marine Biological Resources, Stazione Zoologica Anton Dohrn, Villa Comunale, 80121 Napoli, Italy; luca.ambrosino@szn.it (L.A.); chiusano@unina.it (M.L.C.); 3 Department of Biology and Evolution of Marine Organisms, Stazione Zoologica Anton Dohrn, Villa Comunale, 80121 Napoli, Italy; salvatore.daniello@szn.it; 4 Department of Agriculture, Università degli Studi di Napoli Federico II, 80055 Portici, Italy

**Keywords:** endostyle, thyroid gland, comparative genomics, ascidians, cephalochordates

## Abstract

The specification of the endostyle in non-vertebrate chordates and of the thyroid gland in vertebrates are fundamental steps in the evolution of the thyroid hormone (TH) signaling to coordinate development and body physiology in response to a range of environmental signals. The physiology and biology of TH signaling in vertebrates have been studied in the past, but a complete understanding of such a complex system is still lacking. Non-model species from non-vertebrate chordates may greatly improve our understanding of the evolution of this complex endocrine pathway. Adaptation of already existing proteins in order to perform new roles is a common feature observed during the course of evolution. Through sequence similarity approaches, we investigated the presence of bona fide thyroid peroxidase (TPO), iodothyronine deiodinase (DIO), and thyroid hormone receptors (THRs) in non-vertebrate and vertebrate chordates. Additionally, we determined both the conservation and divergence degrees of functional domains at the protein level. This study supports the hypothesis that non-vertebrate chordates have a functional thyroid hormone signaling system and provides additional information about its possible evolutionary adaptation.

## 1. Introduction

**Figure 1:**
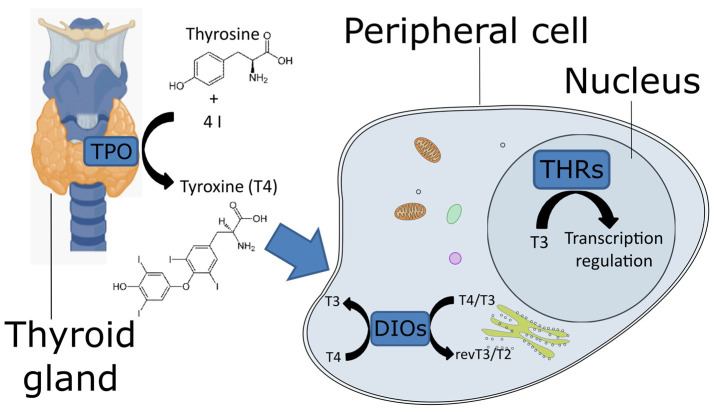
Diagram representing the thyroid hormone signaling system. The thyroid drawing was taken from BioRender (https://biorender.com/, access date 15 September 2021), and the stylized cell was drawn starting from the animal cell clipart (https://svg-clipart.com/cartoon/lCu7ktQ-animal-cell-clipart, access date 15 September 2021).

Via thyroid hormone (TH) signaling, organisms are able to regulate many physiological processes. In animals, this system is very important for key biological features such as development, metamorphosis, and cell and organism regeneration [1,2,3]. Thyroid hormone signaling has emerged as a biological system to convey environmental information to cells and maximize the exploitation of environmental resources. Moreover, it may be considered a key regulator of development and metabolism that arose very early in life history [3]. Over the last decades, thyroid hormone signaling pathways have been deeply characterized mainly in vertebrates. Among the many elements involved in this signaling pathway, there are three key enzymes that have also been identified in a few invertebrate taxa [4]. The study of these three enzymes—thyroid peroxidase (TPO), selenoprotein iodothyronine deiodinase (DIO), and the thyroid hormone receptor (THR)—may unveil the evolutionary route of the TH signaling pathway from non-vertebrate to vertebrate chordates [5]. TPO, located in the thyroid gland, synthesizes the pro-hormone L-thyroxine (known as T4) using iodine and tyrosine as precursors (Figure 1). TPO belongs to the animal peroxidase family [6], which includes large proteins (approximately 700 aa) containing heme groups. This enzyme catalyzes the oxidation of iodine by H_2_O_2_ as an electron acceptor; the oxidized form of iodine reacts with tyrosine to produce T4. The mechanisms of action of peroxidases rely on two histidines (named the distal and the proximal histidines in the work of Taurog [1]), a catalytic arginine, and an asparagine residue, linked to the proximal histidine. These residues, together with the heme iron center, cleave the O=O bond of the hydrogen peroxide to oxidize iodine. The T4 is transported to the peripheral tissues, where DIO catalyzes its conversion into the active form 3,3′,5-triiodothyroxine (known as T3, Figure 1). In vertebrates, DIOs consist of three paralogous proteins that exert slightly different functions to fine-tune the action of T4. They have different catalytic properties and are located in different cellular compartments, where they can be expressed at different stages. DIO1 and DIO2 convert T4 to T3, whereas DIO3, also known as thyroxine 5-deiodinase, converts T4 and T3 into inactive metabolites (reverse T3 and T2, respectively) [7]. DIO1 is present on the plasma membrane, DIO2 on the endoplasmic reticulum [8] and DIO3 can be located on either the plasma membrane or the nuclear membrane [9]. T3 binds the THRs (Figure 1), which belong to the nuclear receptor superfamily 1. These receptors contain a DNA-binding domain at the N-terminal and a ligand-binding domain at the C-terminal. The former is well conserved, whereas the latter exhibits more variability [10]. The genetic modulation mechanism consists of the binding of THR to the thyroid hormone response elements, which are specific short genomic elements. In particular, the THR protein binds as a monomer, homodimer, or heterodimer to regulate the transcription of downstream genes [11].

Knowledge about the thyroid hormone signaling system in non-vertebrate chordates, including cephalochordates and tunicates, is still incomplete, and it is limited to a few studies on some model species [12,13,14,15]. However, in the last decade, scientific evidence has been accumulating that thyroid hormone signaling, homologous to the one known in vertebrates, are present in non-vertebrate chordates as well and probably even outside chordates/deuterostomes [16].

In fact, some evidence from non-vertebrate chordates suggest the involvement of TH signaling in the metamorphosis regulation [2,5]. The presence of T4 in the tunicate *Ciona intestinalis* has been suggested by immunodetection methods [6], while in the cephalochordate (amphioxus) *Branchiostoma lanceolatum*, a smaller derivative of triiodothyroacetic acid (TRIAC) was identified as the primordial bioactive TH [5,7]. DIO and THR vertebrate orthologs have been identified in the tunicates *Halocynthia roretzi* and *C. intestinalis* and in cephalochordates; however, since they are so divergent, it is difficult to clarify their binding activities [6,9]. In amphioxus, there are eight iodine symporter-related genes that may participate to the transport of TH-like compounds [11], suggesting that they may have a role in the metabolism and transport of these active compounds. Nevertheless, as in tunicates, TH binding to THRs has not been demonstrated yet [4,12]. 

Recently, the functional characterization of thyroid-stimulating hormone in cephalochordates and the identification of a thyroid-stimulating hormone orthologue in the genome of tunicates further suggest an ancestral origin of the TH signaling pathway [13]. 

However, the vertebrate model of iodine uptake cannot be generalized in all chordates, and the evolutionary steps that led to complete hormonal control, which on the other hand can be observed in vertebrates, are still an open question.

Cephalochordates and tunicates produce TH in the endostyle, a specialized organ homolog to the vertebrate thyroid gland [14,15]. Interestingly, both the thyroid gland and endostyle are closely associated with the pharyngeal region of the digestive tract, suggesting a link between thyroid hormone function and food uptake [11,14,16].

A noteworthy example of thyroid signaling evolution may be found in the agnathan lamprey, which exhibits at the larval stage of an endostyle, which in the adult stage, after metamorphosis, becomes a thyroid gland-like organ [17]. Thus, it has been suggested that the endostyles of urochordates, cephalochordates, and lampreys are homologous to each other and that they were precursors of the proper thyroid gland, typical of vertebrates (reviewed in [18]).

It is clear that during TH signaling evolution, the ability to self-synthesize TH has been a fundamental acquisition. To decode the functional evolution of this signaling system and to understand its role in chordates before the emergence of a defined thyroid gland, it is fundamental to investigate the evolution of the genetic toolkit involved in the TH signaling among as many species of chordates as possible.

With this purpose, we focused our attention on *TPO*, *DIO*, and *THR* looking for orthologous genes and proteins both in invertebrate and vertebrate chordates, taking advantage of available genome sequences and transcriptome data. We further investigated the presence of specific protein domains and the divergence of their flanking sequences in order to establish their structural properties and their evolutionary conservation.

## 2. Materials and Methods

We obtained the list of the TPO, THRs, and DIOs orthologs in non-vertebrate chordates with the combination of two approaches: (i) we created a reference data set of proteins from annotated collections; (ii) we increased the data set by including computationally defined homolog proteins. More specifically, in the first step, the sequences representing TPO, DIO, and THR proteins were downloaded from the UniProt database [19] including those from exemplificative vertebrates. With this aim, we queried the InterPro public resource [20] using TPO, DIO, and THR reference sequences from *Homo sapiens* and retrieved the associated family and domain entries. All UniProt proteins (both reviewed and unreviewed) deriving from non-vertebrate chordates and associated to the detected entries (i.e., IPR019791 (“thyroid peroxidase”), IPR000643 (“iodothyronine deiodinase”), and IPR001728 (“thyroid hormone receptor”)) were downloaded in the FASTA format, while the corresponding vertebrate entries were selected from the reviewed collection. Among vertebrates, we retained data on the model species *H. sapiens*, *Gallus gallus*, *Danio rerio*, and *Petromyzon marinus*. The vertebrate sequences, if not available in UniProt, were obtained by querying the NCBI protein database [21]. When multiple sequences of the same protein in the same species were available, we performed a BLAST sequence similarity search [22] selecting the highest similar ones with respect to the human protein. In the second step, we searched for ortholog sequences of the human reference proteins by performing BLAST similarity searches [22] against either the available proteome or transcriptome from the ANISEED [23] collection for those species that had missing data in the UniProt database.

We considered the non-chordate deuterostome—the purple sea urchin *Strongylocentrotus purpuratus*—as the outgroup species in the phylogenetic analyses. Specifically, the outgroup for the TPO sequence analysis was represented by the homologous protein ovoperoxidase as was conducted in previous works [24]. The outgroup protein for DIOs was the iodothyronine deiodinase (acc. no. A0A7M7HMF2), while for THRs, it was the thyroid hormone receptor (acc. no. XP_030829710.1). For each protein, the homologous sequences across all species were aligned using the MAFFT software [25]. The sequences in the multiple alignments were trimmed to remove background noise using the trimAl software [26] with the following options “-gt 0.25 –res overlap 0.25 –seq overlap 75”. Phylogenetic trees were reconstructed using the maximum likelihood method, utilizing the LG model for aminoacidic substitution [27] and calculating a bootstrap after 999 permutations. These analyses were performed using the software MEGA X (Molecular Evolutionary Genetics Analysis across computing platforms) [28]. The phylogenetic tree “branches” length represents the number of substitutions per site, which is a standard measure. With this scale, it is possible to estimate the number of substitutions occurring between two given leaves of a tree by summing the length of the branches and multiplying it by the length of the alignment. In order to identify conserved motifs, aligned sequences were submitted to the online tool MEME suite [29]. 

## 3. Results and Discussion

### 3.1. The Search for the TH Protein Members and Removal of Misannotated Sequences

The InterPro public resource contains 207 sequences associated to the query IPR019791 TPO. Among vertebrates, the sequences from *H. sapiens*, *D. rerio*, *P. marinus*, and *G. gallus* were retained for further analyses. Among the unreviewed proteins from non-vertebrate chordates, eight sequences were annotated as TPO: one was from *C. intestinalis*, five from *Branchiostoma belcheri*, and two from *Branchiostoma floridae*.

We obtained for IPR000643 (“iodothyronine deiodinase”) 64 sequences, and 62 could be annotated as type I or II DIO or thyroxine 5-deiodinase (i.e., DIO1, DIO2, and DIO3, respectively). The two remaining sequences, annotated as “uncharacterized protein”, were removed as well as the DIO proteins from vertebrates other than *H. sapiens*, *P. marinus*, *G. gallus*, and *D. rerio*. This procedure resulted in 43 sequences. All three DIO paralogues were retrieved for *H. sapiens, G. gallus*, and *D. rerio*. A single DIO sequence was retrieved for *P. marinus*, showing the highest similarity score with the human DIO2 (46% identities and 59% positives). The orthologous sequence of DIO3 for this species was retrieved from the NCBI protein database. The remaining sequences belonged to *B. belcheri* (nine sequences), *B. floridae* (seven sequences), *Oikopleura dioica* (eight sequences), *C. intestinalis* (two sequences), and *H. roretzi* and *Phallusia mammillata* (one sequence each).

The search for IPR001728 (“thyroid hormone receptor”) produced 97 sequences. Similar to TPO, many proteins have generic annotations such as “nuclear receptor” or “LBD domain-containing protein”. We kept those proteins with a functional annotation corresponding to “THR” in non-vertebrate chordates and orthologous of the vertebrate species (two paralogous from *H. sapiens*, *G. gallus*, and *P. marinus* and four paralogues from *D. rerio*). In addition to the THRs, for the reference vertebrate species, the sequences retrieved for non-vertebrate chordates were from *B. belcheri*, *B. floridae*, and *Branchiostoma lanceolatum* (one sequence for each species).

We further investigated the ANISEED resource by sequence similarity, looking for TH orthologue sequences in tunicates that were missing from the previous step. Thanks to this approach, we detected 15, 22, and 20 more sequences for TPO, DIOs, and THRs, respectively (Table 1).

### 3.2. Thyroid Peroxidase

A single protein from the TPO class was found in all of the species here considered (Supplementary Materials File S1). The protein sequence lengths for both vertebrate and non-vertebrate chordates ranged from 549 aa (for *P. mammillata*) to 1415 aa (for *B. belcheri*); the second longest sequence was from *M. oculata* (970 aa long) and resulted in an alignment of 1651 positions (Supplementary Materials Figure S1). The trimmed alignment consisted of 962 phylogenetically informative sites (Supplementary Materials Figure S2). The aligned sequences showed an average identity of 34.79% ± 11.08%, ranging from 18.2% (in the comparison between *B. belcheri* and *Phallusia fumigata*) to 82.3% (among the two *Halocynthia* species).

**Figure 2:**
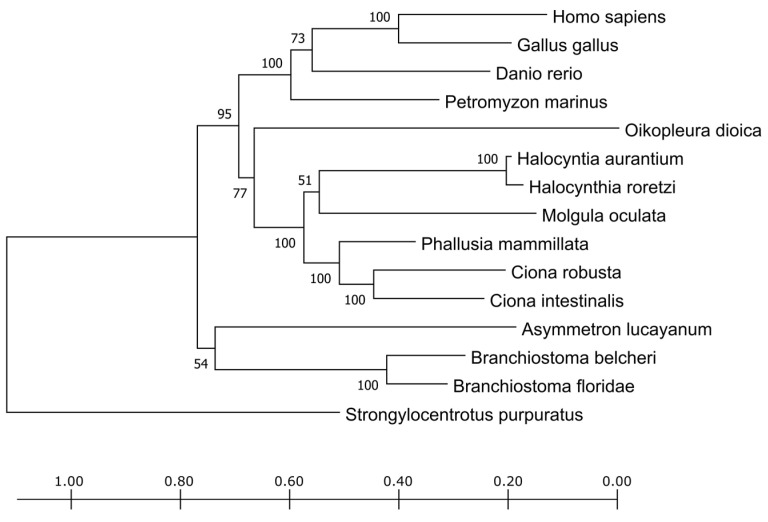
Phylogenetic tree of TPO proteins inferred by the maximum likelihood method. Pictured is the tree with the highest log likelihood (−20,923.40). The percentage of trees in which the associated taxa clustered together is shown next to the branches. Initial tree(s) for the heuristic search were obtained automatically by applying the neighbor-join and the BioNJ algorithms to a matrix of pairwise distances estimated using the JTT model as implemented in the software MEGA X [28] and then selecting the topology with best log likelihood value. At the bottom, there is the substitution per site, a standard measure for tree branch length. A total of 956 positions were considered in the alignment.

The phylogenetic tree obtained from the trimmed alignment of the TPO sequences is shown in Figure 2. The vertebrates form a distinct cluster as well as the tunicates and cephalochordates, reflecting their divergence in the chordate phylum evolution. The sequence deriving from *P. fumigata* was too short, lacking most of the amino acid sites, and, from a preliminary screening, it clustered together with cephalochordates. The short length is probably due more to the fact of poor annotation (e.g., due to the fact of assembly mistakes or low reads coverage) rather than to a real biological variant. To avoid inaccurate contributions of this sequence to the alignment, it was eliminated from the phylogenetic tree shown in Figure 2. 

**Figure 3:**
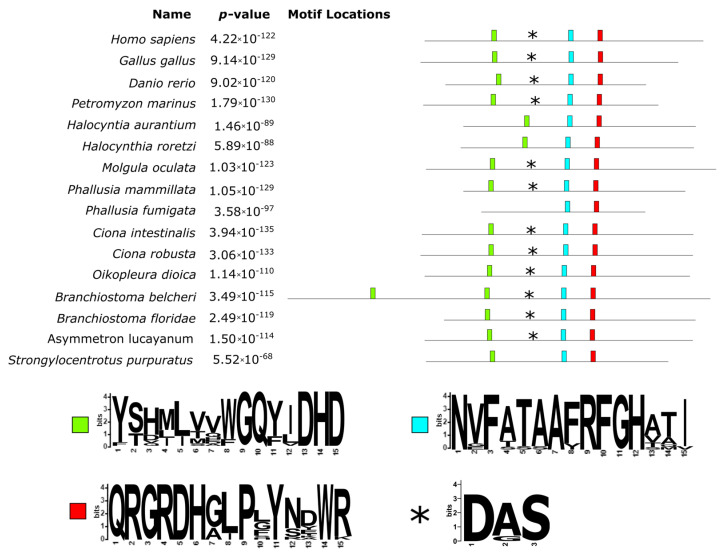
Conserved domain locations and SeqLogo as inferred by the MEME suite on the TPO protein sequences. The proteins were aligned to highlight the occurrence and positioning of the homologous conserved domains; the positions of the boxes reflect the actual positions in the alignment (see Supplementary Materials Figure S1). Distal and proximal histidine conserved domains are shown in the green and light blue boxes, respectively, while the asterisks denote the region of the Ca-binding site. Slightly downstream, the third conserved sequence containing the Asn residue can be found (red box).

As shown in Figure 3, the alignment of TPO proteins underlined a large insertion (383 aa long, from position 271 to 654 of the alignment) in the protein of *B. belcheri* and two 60 aa long deletions for both *Halocynthia* species (located at positions 772–849 and 938–997 of the alignment). Interestingly, the analysis of the conserved motifs revealed that the long insertion in the *B. belcheri* protein contained a second copy of the conserved motif GQ(Y/F)(I/L/V)DHD (green box in Figure 3), which according to Taurog, 1999 [1] contains a distal histidine residue. The observed differences in these two sites indicate that they were likely derived from a duplication event that only took place in the *B. belcheri* genome. A proximal histidine was also well conserved (light blue box in Figure 3) and represented by the motif RFGH (positions 1039–1043 of the alignment). Another well conserved site had the motif QRGRDH (position 1132–1137 of the alignment), located immediately downstream of the conserved Asn residues (red box in Figure 3), which form hydrogen bonds with the proximal histidine. A closer look at the two *Halocynthia* spp.’s first deletion revealed that it includes an homologous region to the Ca-binding site (asterisks in Figure 3). Downstream of this deletion, there was a conserved motif with the pattern VYGS.

### 3.3. Iodothyronine Deiodinase

**Figure 4:**
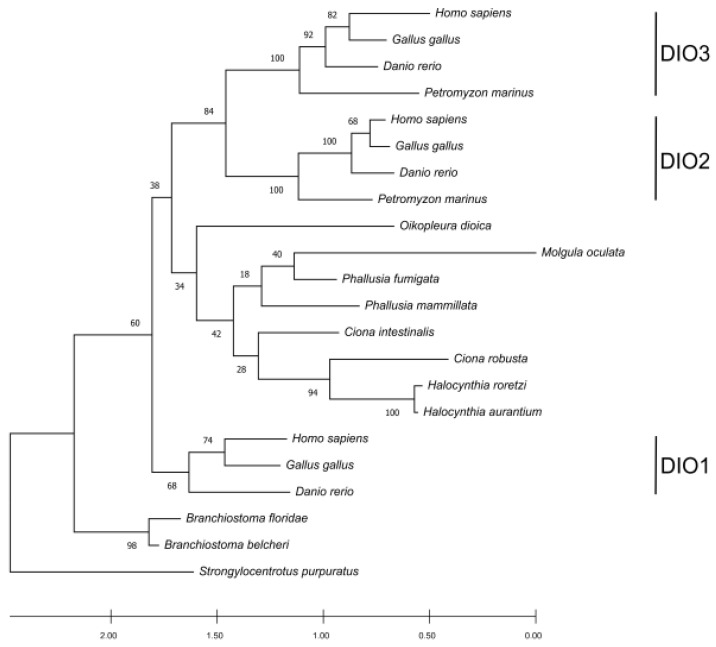
The phylogenetic tree inferred using DIO amino acid sequences (22 sequences aligned on 274 positions). The tree was built via the maximum likelihood method using the JTT model for aminoacidic substitution as implemented in the software MEGAX [28]. The tree with the best log likelihood (−7865.03) is shown. The bootstrap support is shown on each node. The scale at the bottom is the substitution per site, a standard measure of tree branch length.

Searching for DIO orthologs in both non-vertebrate chordates and *P. marinus*, we identified only two out of three paralogues in *P. marinus* and a single homolog protein in non-vertebrate chordates except for *A. lucayanum*, where none could be detected (Supplementary Materials File S2). The sequence length for the DIO orthologs ranged from 118 aa (for *H. aurantium*) to 305 aa (for the H. sapiens DIO3) and resulted in the alignment of 424 positions (Supplementary Materials Figure S3). The alignment was trimmed and found to consist of 273 phylogenetically informative sites (Supplementary Materials Figure S4). These sequences showed an average identity of 24.65% ± 10.79%, ranging from 11.9% (with regard to the comparison between *O. dioica* and *M. oculata*) to 82.3% (for that which concerns the two DIO2 paralogues from *H. sapiens* and *G. gallus*). The phylogenetic tree displayed a clustering pattern that grouped the three paralogues together with the vertebrates, supported by high bootstrap support (Figure 4). Previous studies suggest that DIO1 is the oldest among the paralogues in vertebrates, and that DIO2 and DIO3 are the most recent ones [30]. The phylogenetic tree confirms that DIO1 proteins show a high conservation degree and form a cluster outside an ingroup represented by DIO2 and DIO3 subfamilies. Tunicate DIOs form a cluster that branches as a sister group with the DIO2 and DIO3 paralogues in vertebrates. Looking at the positions of the tunicate DIOs in the tree, there could be some discrepancies with the known species phylogeny. This could be explained by the high sequence divergence among the analyzed species. However, the low bootstrap support for the clustered branches of the tree can help in addressing the main relationships. When the bootstrap value is low, there could be sites missing from an absent protein portion, as it can be observed in most tunicates (Supplementary Materials Figures S3 and S4); alternatively, the tree could be produced by the homoplasy generated by a data set consisting of a different number of paralogues among the taxa. Considering the bootstrapping value derived from vertebrates together with the position of the single-copy DIO orthologs in tunicates, it becomes evident that this clade groups independently from vertebrates, while the vertebrate DIO2/3 paralogs appear as more recent and vertebrate specific. The placement of *Branchiostoma* spp. as an outgroup of all the proteins (including the DIO1 cluster) could be explained by a higher conservation caused by the *Branchiostoma* evolutionary rate being one of the slowest in non-vertebrate chordates [31]. The absence of DIO proteins in *Asymmetron lucayanum* is likely due to the lack of genome information. Alternatively, but less likely, this gene has been lost as a synapomorphy in this species, which could have kept relying on the TRIAC as a thyroid hormone signaling system analogous pathway. The biosynthetic pathway described for the amphioxus TRIAC, which is characterized by an inner ring deiodination catalyzed by a non-selenoprotein DIO that acts selectively and does not show activity towards T3 and T4 [7], could support the second hypothesis [7]. Consequently, both of these hypotheses require further explorations. As expected from the 2R genome duplication hypothesis, the number of DIOs found in non-vertebrate chordates is lower than the ones in vertebrates. Since DIOs are essential for regulating the active/non-active thyroid hormone ratio at the peripheral level, it seems that the genes involved in this regulatory pathway were duplicated in concomitance with the emergence of anatomically and histologically more complex organizations. This evidence is also consistent with the limited number of paralogues found in *P. marinus*, which features a more complex design compared to the amphioxus but not as complicated as the one in vertebrates.

**Figure 5:**
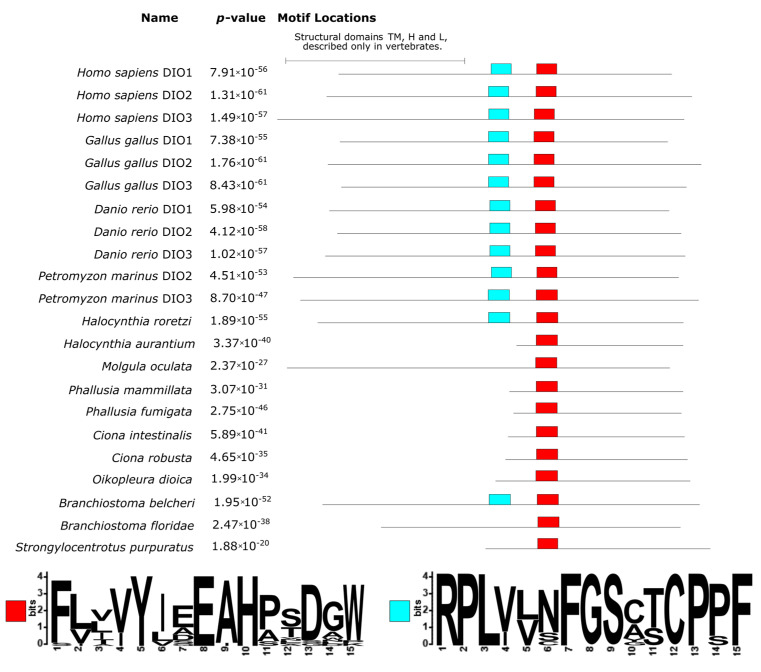
Conserved domain locations and SeqLogo as inferred by the MEME suite on the DIO protein sequences. The proteins were aligned to highlight the occurrence and positioning of the homologous conserved domains, the positions of the boxes reflect the actual positions in the alignment (see Supplementary Materials Figure S3). The ubiquitous red box domain corresponds to the dimerization domain, whereas the light blue box is the catalytic globular domain G as described by Orozco et al. (2012) [30].

The three DIOs show a similar organization in vertebrates, consisting of four domains: an N-terminal transmembrane domain (TM), a hinge (H), a linker (L), and a globular domain with catalytic function (G) [30]. The latter is the most conserved one, whereas the former three are located in the region at the N-terminal, which is more variable [30]. Another conserved domain, according to Orozco et al. (2012), is implicated in the deiodinase dimerization domain [30]. The proteins from non-vertebrate chordates, however, apparently do not possess TM, H, and L domains. These proteins were generally shorter than the ones from vertebrates. In particular, the sequences from the two *Ciona* and *Phallusia*—*H. aurantium* and *O. dioica*—were missing almost half of the protein sequence at the N-terminal (Figure 5). The analysis of conserved domains found two conserved domains, one with the motif RPLYXXFGS (light blue box in Figure 5) that corresponded to the G domain in vertebrates. Another conserved domain was located downstream and had the motif (V/I)YXEAH (red box in Figure 5), corresponding to the dimerization domain of vertebrates. While the former was not found in all species, the latter was ubiquitous. Interestingly, the RPLYXXFGS motif was found in all vertebrates in *H. roretzi* and *B. belcheri* and, hence, the presence of an active homologue of the vertebrates iodothyronine deiodinase was, for the first time, experimentally demonstrated in non-vertebrate chordates precisely for the ascidian species *H. roretzi* [9]. Another question mark is the absence of the G domain in all tunicate species and two out of three cephalochordates. This suggests that the G domain is crucial for protein functioning in vertebrates, but the homologous proteins in protochordates may possess different catalytic sites. Further investigation of the structural gene annotation of tunicates is required to deeply investigate this aspect.

### 3.4. Thyroid Hormone Receptors 

We found two THR proteins for most vertebrates (in *D. rerio* there were four), whereas only one in all non-vertebrate chordates was here analyzed (Supplementary Materials File S3). These proteins’ length ranged from 315 aa (for *P. fumigata*) to 592 aa (*C. robusta*). The alignment had 1295 positions, explained by the two *Halocynthia* sequences which had lengths over 1000 aa each (Supplementary Materials Figure S5). Sequences’ identities were, on average, 40.81% ± 22.18%, ranging from a minimum of 11.8% (deriving from the comparison between *P. mammillata* and *C. robusta*) to a maximum of 99.6% (for what concerns the two *Phallusia* species). The trimmed alignment resulted in 443 phylogenetically informative positions (Supplementary Materials Figure S6).

**Figure 6:**
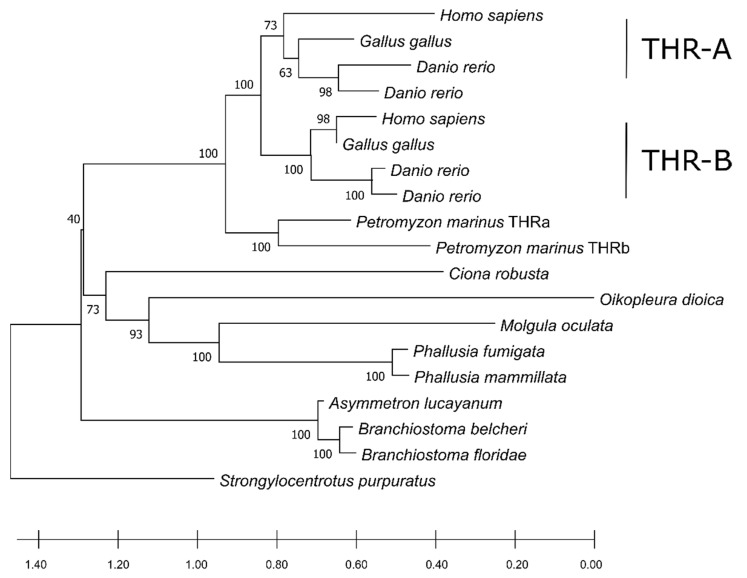
The phylogenetic tree inferred from THR sequences using the maximum likelihood method. The tree with the highest log likelihood (−8096.50) is shown. The percentage of trees in which the associated taxa clustered together is shown next to the branches. The initial tree(s) for the heuristic search were obtained automatically by applying the neighbor-join and BioNJ algorithms to a matrix of pairwise distances estimated using the JTT model as implemented in the software MEGAX [28] and then selecting the topology with the best log likelihood value. The scale at the bottom is the substitution per site, a standard measure of tree branch length. There was a total of 443 positions in the final data set.

The phylogenetic tree shows a distinct clustering pattern in the vertebrates, separating the two paralogues in a similar fashion to the DIOs. *C. robusta* constituted an independent-one-taxon branch from the remaining group of Ascidiacea, where *Branchiostoma* spp. was the outgroup of other chordates (Figure 6). Moreover, the two THR paralogues in *P. marinus* had a similarity score of 68.4% and grouped together but did not cluster with neither the vertebrates THRa nor THRb. This fact could point to a separate duplication event that gave rise to two copies of THR and probably occurred in the common ancestor of lampreys. We detected a single ortholog in non-vertebrate chordates in THRs as well. This suggests that the duplication of the THR is also associated with the emergence of a more complex physiology and anatomy of vertebrates, i.e., THRs required a more subtle modulation of gene expression in different moments of metamorphosis in *Xenopus laevis* [32]. This further confirms that a higher number of paralogues in this regulatory pathway led to greater tissue diversification in vertebrates.

**Figure 7:**
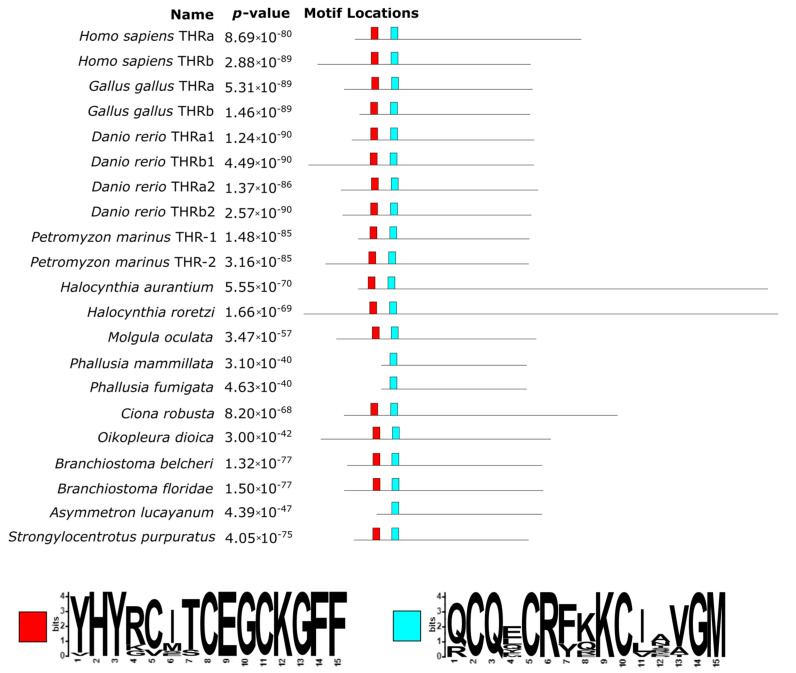
Conserved domain locations and SeqLogo as inferred by the MEME suite on the THR protein sequences. The proteins were aligned to highlight the occurrence and positioning of the conserved domains. The position of the boxes reflects the actual positions in the alignment (see Supplementary Materials Figure S5). The N-terminal DNA-binding domain contained two conserved sequences, a P-box motif (red) and a T-box/A-box motif (light blue).

In comparison to both TPOs and DIOs, THRs are better conserved, since fewer large re-arrangements were detected, i.e., a 66 aa long insertion (positions 629–695 of the alignment) in the human THR alpha and an extended C-terminal portion in the two *Halocynthia* species (Figure 7; Supplementary Materials Figure S5).

The N-terminal region of these sequences contains different domains related to DNA binding such as the DNA-binding domain and the P-box. While the former was well conserved across all sequences, including basal chordates (Figure 7), the latter was not present in the two *Phallusia* species (red box in Figure 7), and only *B. belcheri* contained a D residue instead of an E, which is crucial for DNA-binding specificity. As shown in Figure 7 (light blue box), 30 amino acids downstream of a second conserved region, corresponding to a T-box and an A-box, can be found. These two regions contain a zinc-finger domain, involved in the recognition of the DNA-binding site, and are crucial in the dimerization of the protein [33]. 

## 4. Conclusions

The presence of a thyroid hormone-producing endostyle in non-vertebrate chordates supports the hypothesis that the thyroid hormone signaling system is an ancient feature of all chordates. Homologous proteins for each of the three key proteins in the thyroid hormone signaling system were found for most of the tunicates and cephalochordates. However, we detected fewer paralogous genes for DIO and THRs. We discovered a duplication for a conserved domain in the TPO of *B. belcheri* and highlighted the possible evolutionary framework of the DIO proteins. Such proteins displayed higher conservation in cephalochordates compared to the tunicates and evolved by two runs of duplications exclusively in vertebrates, in agreement with the 2R hypothesis. Our analyses also highlighted the absence of a conserved catalytic globular domain G in most but not all the non-vertebrate chordate DIOs. Better structural annotation of the genomes of tunicate and amphioxus species will help to check the real loss of this domain and the possible presence of a catalytic site with different functional characteristics. For that which concerns THRs, we propose that two independent duplication events occurred that led to the two THR paralogues in vertebrates. A first gene duplication took place in the ancestor lineage leading to *P. marinus* and a second one in the common vertebrate ancestor. In the former species, the divergence among the two paralogues was lower than in the other vertebrates. Our findings further corroborate the fundamental role played by large-scale gene duplications in vertebrates after the split between them and the other chordates thus allowing for the development of more complex regulatory mechanisms in the thyroid hormone signaling pathway and, accordingly, promoting a more extensive and specialized tissue diversification. 

## Figures and Tables

**Table 1:** Summary of the sequences retrieved from the UniProt database or inferred by sequence similarity searches; all sequences can be found in Supplementary Material Files S1–S3.

Taxon	Species	TPO	THRs		DIOs		
Paralogues		Single Paralogue in All Species	THRa	THRb	DIO1	DIO2	DIO3
Vertebrates	*Homo sapiens*	P07202	P10827	P10828	P49895	Q92813	P55073
Vertebrates	*Gallus gallus*	XP_040523114.1	P04625	P68306	O42411	Q9IAX2	O42412
Vertebrates	*Danio rerio*	XP_0213229452	Q98867,U3JAT9	Q9PVE4,S6BPQ3	NP_001007284.3	NP_997954.4	NP_001171406
Vertebrates	*Petromyzon marinus*	XP_0328815489.1	Q2PK09,Q2PK08		-	L7WGA7	XP_032820886.1
Tunicates	*Ciona robusta*	KH.C8.354.v1.R.ND1-1	KY.Chr11.1143.v1.ND1-1		KY.Chr9.332.v1.SL1-1		
Tunicates	*Ciona intestinalis*	Q9XXZ7	-		H2Y0H0		
Tunicates	*Halocynthia roretzi*	Harore.CG.MTP2014.S42.g15904.01.t	Harore.CG.MTP2014.S302.g07324.01.t		Q6U6H1		
Tunicates	*Halocynthia aurantium*	Haaura.CG.MTP2014.S222.g03436.01.t	Haaura.CG.MTP2014.S16.g00477.01.t		Haaura.CG.MTP2014.S697.g06289.01.t		
Tunicates	*Molgula oculata*	Moocul.CG.ELv1_2.S84209.g06362.01.t	Moocul.CG.ELv1_2.S59439.g03711.01.t		Moocul.CG.ELv1_2.S94311.g07746.01.t		
Tunicates	*Phallusia fumigata*	Phfumi.CG.MTP2014.S1776.g04105.01.t	Phfumi.CG.MTP2014.S839.g02838.01.t		Phfumi.CG.MTP2014.S7504.g07770.03.t		
Tunicates	*Phallusia mammillata*	Phmamm.CG.MTP2014.S185.g04959.01.t	Phmamm.CG.MTP2014.S30.g01201.01.t		A0A6F9DAE7		
Tunicates	*Oikopleura dioica*	Moocul.CG.ELv1_2.S84209.g06362.01.t	Oidioi.CG.OSKA2016.S6.g07336.01.t		Oidioi.CG.OSKA2016.S2.g03083.01.t		
Cephalochordates	*Asymmetron lucayanum*	gb|GETC01130702.1	gb|GETC01035308.1		-		
Cephalochordates	*Branchiostoma floridae*	C3YBC9	A7L5U9		C4A0H0		
Cephalochordates	*Brachiostoma belcheri*	A0A6P4ZNS4	A0A6P4ZQY8		XP_019647376.1		
Echinodermata	*Strongilocentrosus purpuratus*	XP_030847933.1	XP_030829710.1		A0A7M7HMF2		

## Data Availability

The sequence data reported in the results can be found in the public databases UniProt (https://www.uniprot.org, access date 15 September 2021), NCBI (https://www.ncbi.nlm.nih.gov, access date 15 September 2021), ANISEED (http://www.aniseed.cnrs.fr/, access date 15 September 2021), and InterPro (https://www.ebi.ac.uk/interpro/, access date 15 September 2021).

## Supplementary Materials

The following are available online at https://www.mdpi.com/article/10.3390/cells10123391/s1, Figure S1, Alignment of TPO full protein sequences; Figure S2, Alignment of TPO trimmed protein sequences; Figure S3, Alignment of DIO full protein sequences; Figure S4, Alignment of DIO trimmed protein sequences; Figure S5, Alignment of THR full protein sequences; Figure S6, Alignment of THR trimmed protein sequences; File S1, FASTA sequences of the retrieved TPO proteins; File S2, FASTA sequences of the retrieved DIO proteins; File S3, FASTA sequences of the retrieved THR proteins.
