# Supplementary figures and images for: Evolutionary Adaptation of the Thyroid Hormone Signaling Toolkit in Chordates
Source: Cells. 2021 Dec 2;10(12):3391. doi: 10.3390/cells10123391 (PMC8699336; doi:10.3390/cells10123391)

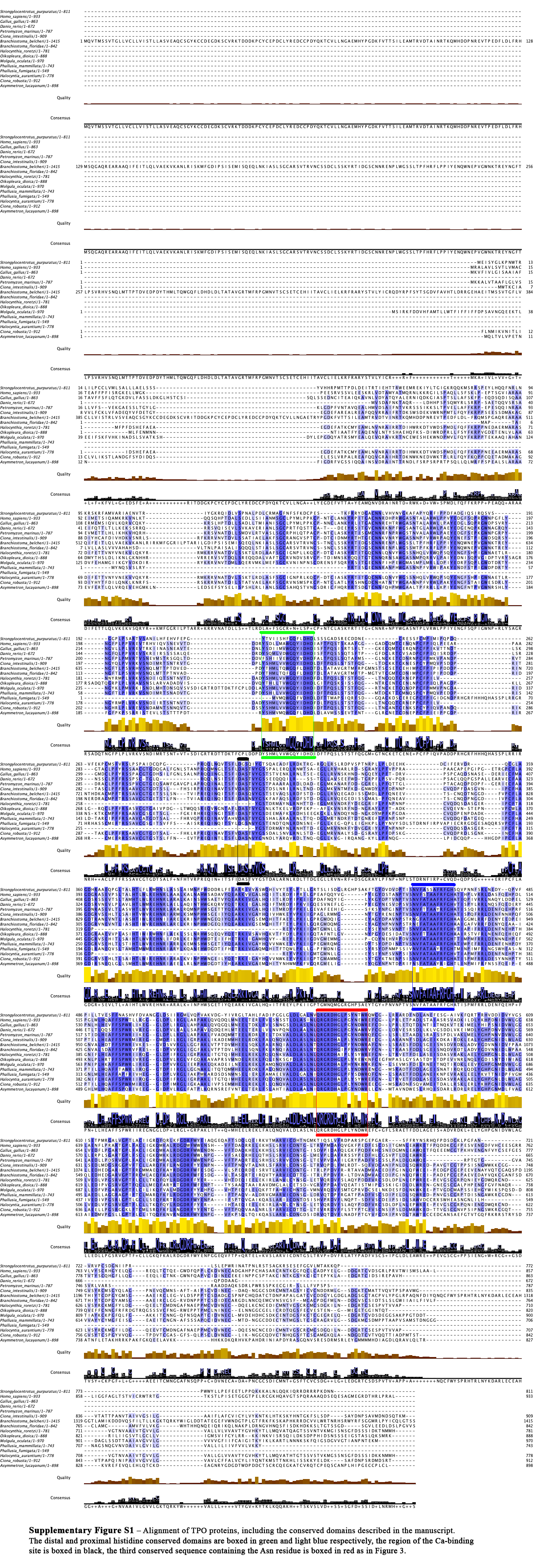

Supplement: Supplementary file 1 [file cells-10-03391-s001.zip › Supplementary Figure S1.tif]

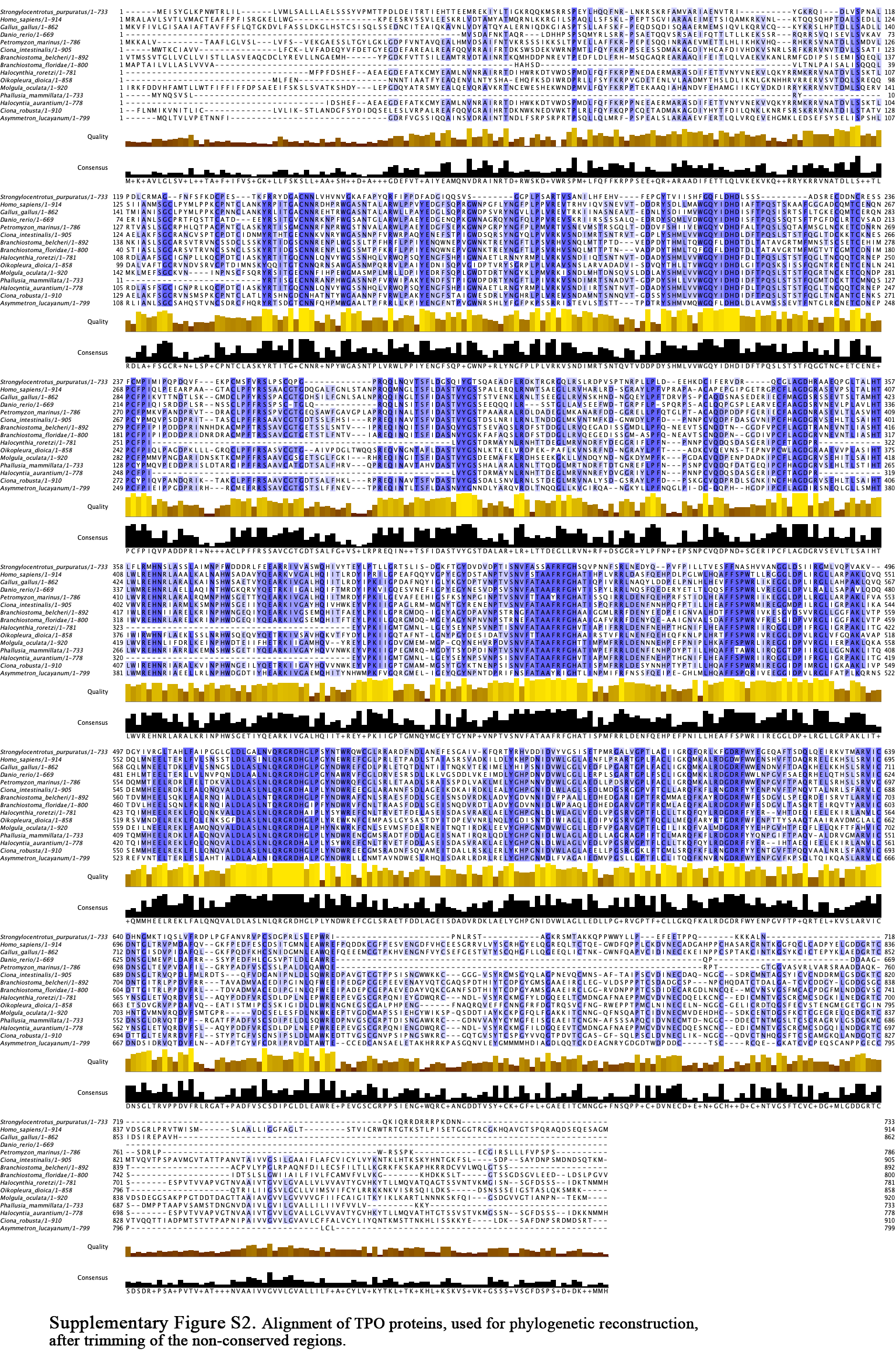

Supplement: Supplementary file 1 [file cells-10-03391-s001.zip › Supplementary Figure S2.tif]

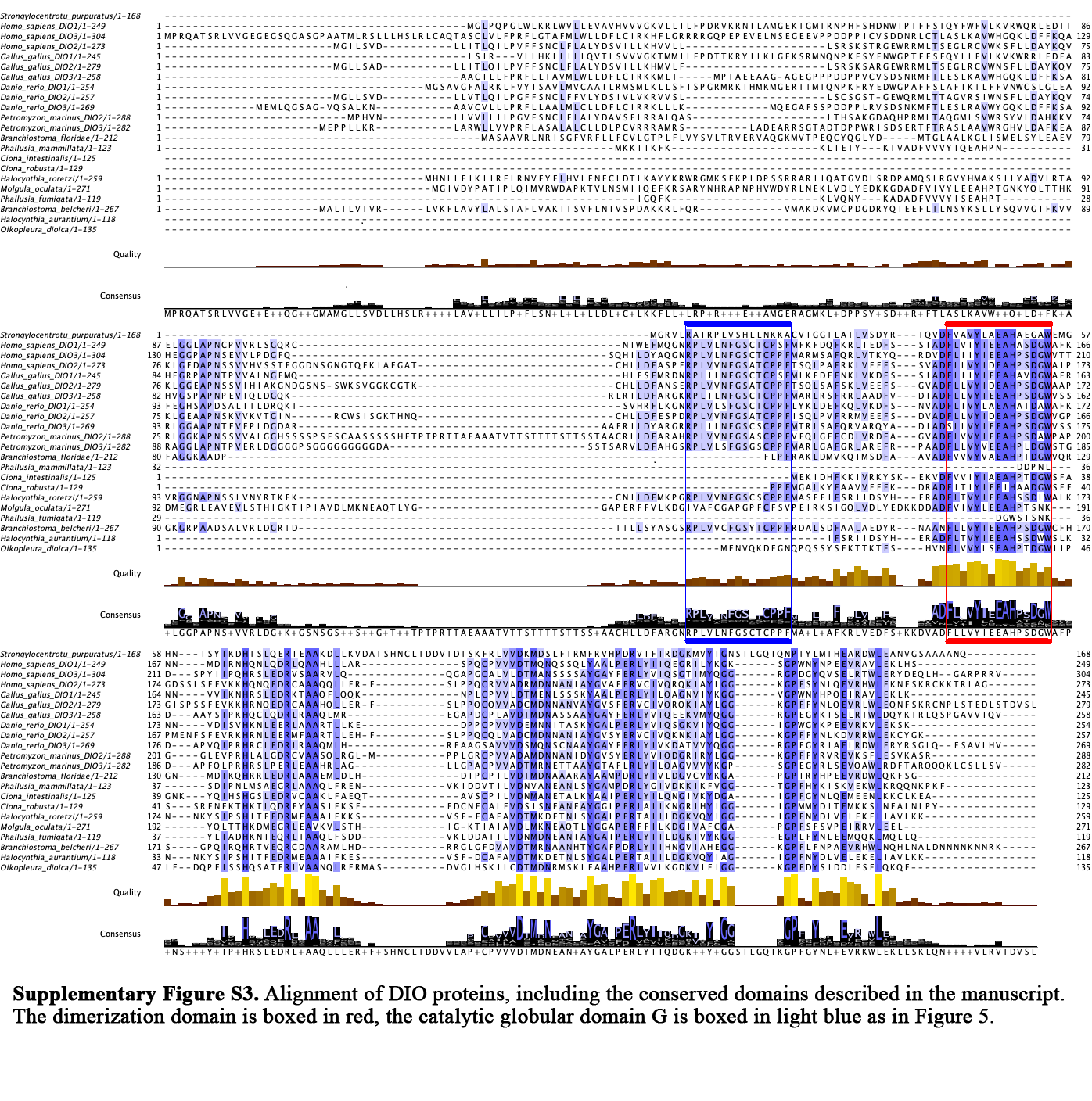

Supplement: Supplementary file 1 [file cells-10-03391-s001.zip › Supplementary Figure S3.tif]

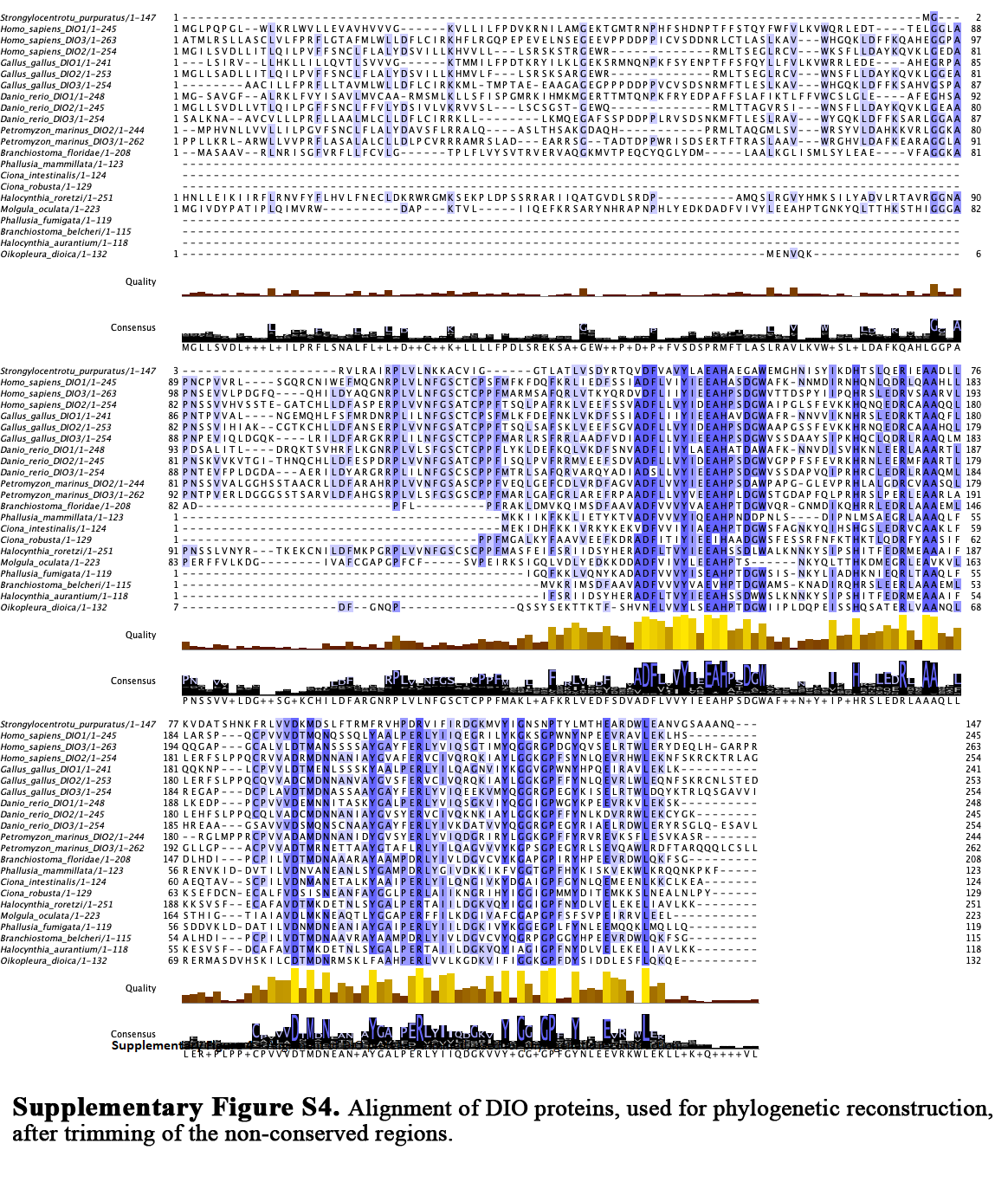

Supplement: Supplementary file 1 [file cells-10-03391-s001.zip › Supplementary Figure S4.tif]

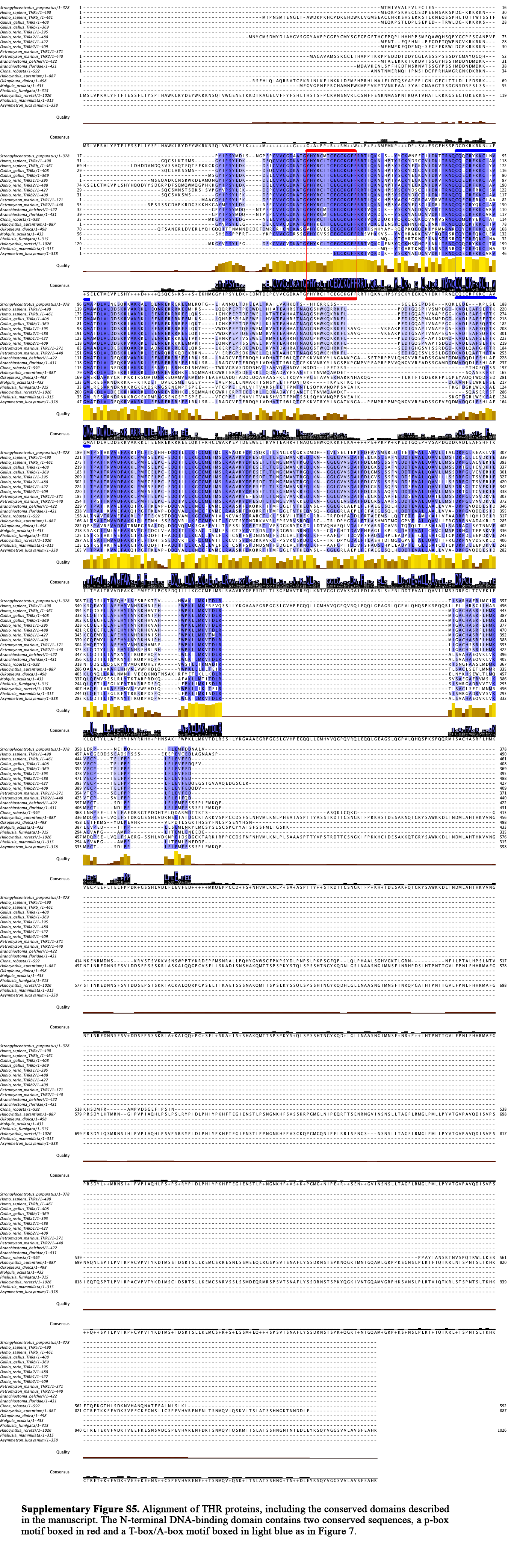

Supplement: Supplementary file 1 [file cells-10-03391-s001.zip › Supplementary Figure S5.tif]

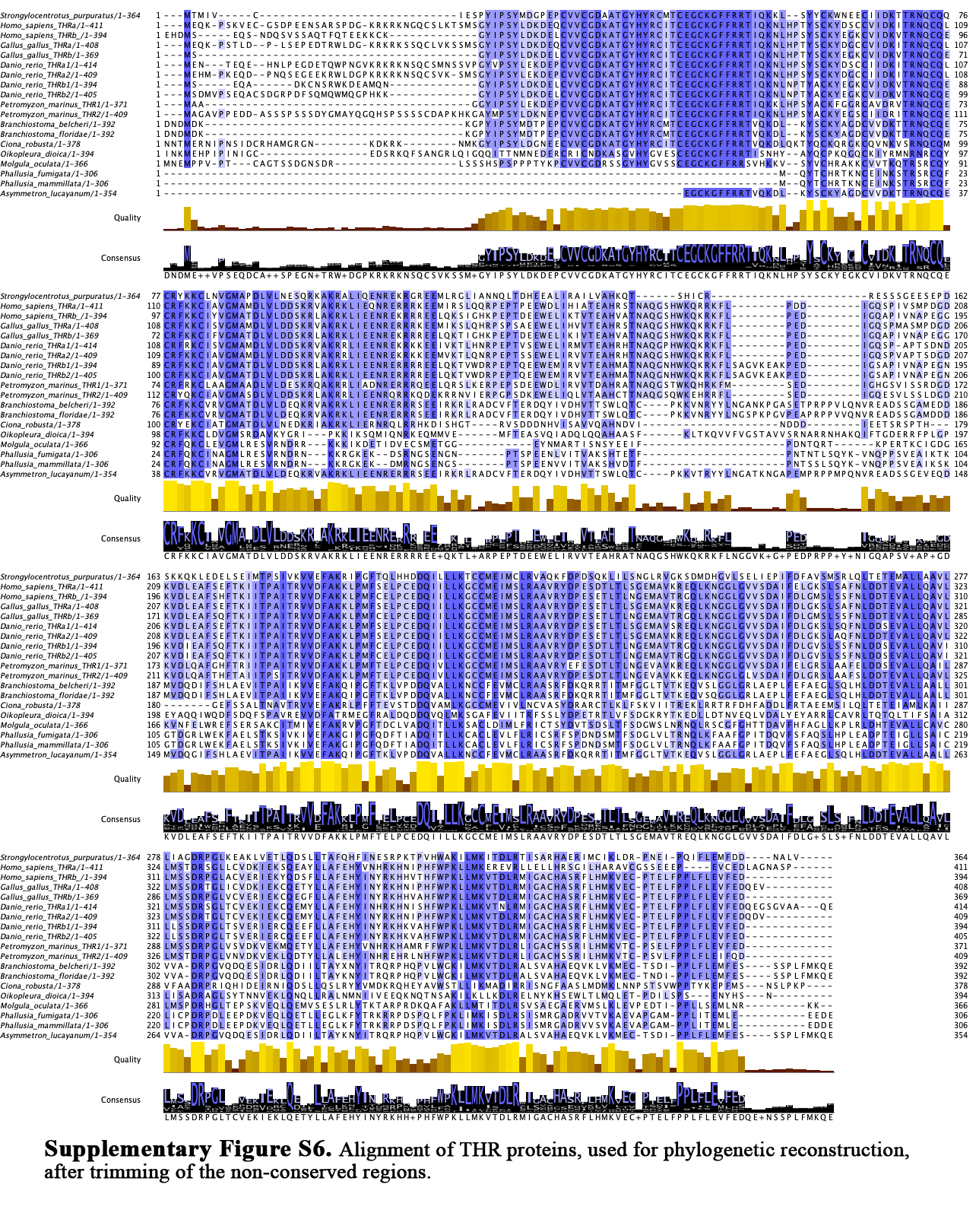

Supplement: Supplementary file 1 [file cells-10-03391-s001.zip › Supplementary Figure S6.tif]
